# The association of Coronavirus Disease-19 mortality and prior bacille Calmette-Guerin vaccination: a robust ecological analysis using unsupervised machine learning

**DOI:** 10.1038/s41598-020-80787-z

**Published:** 2021-01-12

**Authors:** Nathan A. Brooks, Ankur Puri, Sanya Garg, Swapnika Nag, Jacomo Corbo, Anas El Turabi, Noshir Kaka, Rodney W. Zemmel, Paul K. Hegarty, Ashish M. Kamat

**Affiliations:** 1 Department of Urology, The University of Texas MD Anderson Cancer Center, Houston, TX USA; 2 McKinsey & Company, Gurgaon, Haryana India; 3 QuantumBlack a McKinsey Company, London, UK; 4 McKinsey & Company, Waltham, MA USA; 5 McKinsey & Company, Mumbai, Maharashtra India; 6 McKinsey & Company, New York, NY USA; 7 Department of Urology, Mater Private Hospital, Cork, Ireland

**Keywords:** Adjuvants, SARS-CoV-2, Epidemiology

## Abstract

Population-level data have suggested that bacille Calmette-Guerin (BCG) vaccination may lessen the severity of Coronavirus Disease-19 (COVID-19) prompting clinical trials in this area. Some reports have demonstrated conflicting results. We performed a robust, ecologic analysis comparing COVID-19 related mortality (CRM) between strictly selected countries based on BCG vaccination program status utilizing publicly available databases and machine learning methods to define the association between active BCG vaccination programs and CRM. Validation was performed using linear regression and country-specific modeling. CRM was lower for the majority of countries with a BCG vaccination policy for at least the preceding 15 years (BCG15). CRM increased significantly for each increase in the percent population over age 65. A higher total population of a country and BCG15 were significantly associated with improved CRM. There was a consistent association between countries with a BCG vaccination for the preceding 15 years, but not other vaccination programs, and CRM. BCG vaccination programs continued to be associated with decreased CRM even for populations < 40 years old where CRM events are less frequent.

## Introduction

The severe acute respiratory syndrome coronavirus-2 (SARS-CoV-2) and the resulting clinical condition coronavirus disease (COVID-19) have caused a worldwide pandemic. There have been 4.8 million confirmed infections and 318,000 deaths worldwide at the time of analysis on May 19, 2020^1^ resulting in significant global and personal insecurity^2,3^. Mitigation of the pandemic requires a multifaceted strategy to reduce clinical morbidity/mortality, prevent disease spread, and, ultimately, the development of an effective vaccine. Many promising therapies for COVID-19 have demonstrated limited efficacy and the development of a vaccine will take time^4,5^. Supplementing the existing armamentarium for COVID-19 is therefore of the utmost importance.

The bacille Calmette-Guerin (BCG) vaccine has been administered to almost 4 billion people worldwide for nearly 100 years for the prevention of tuberculosis (TB)^6^. Effectiveness for preventing pulmonary TB ranges from 40 to 60% and serious adverse events related to vaccination approach zero^7^. The BCG vaccine is associated with several favorable effects including a reduction in neonatal mortality from respiratory infections and sepsis^8^ as well as in the treatment of bladder cancer^9^. When given in conjunction with anti-viral vaccinations including yellow fever and influenza, patients pre-treated with the BCG vaccine have demonstrated reduced viremia, decreased levels of circulating cytokines associated with cytokine storms, and no difference in, or an improved, anti-viral antibody response^10,11^. These observations may be associated with a shift in the T-cell mediated response to pathogens, enhanced trained innate immunity, and/or an as yet undiscovered pathway^12^. However, they provide an immunologic foundation which suggests BCG vaccination is associated with clinically meaningful immunomodulatory function.

Hegarty and colleagues described the association of the crude case fatality rate (CFR) between 179 total countries with active BCG vaccination programs and those without such programs^13^. The CFR was 0.08 vs 34.8 per million for countries with and without BCG vaccination programs, respectively. In concert with the potential mechanisms described above, this work suggested that BCG vaccination might be associated with decreased COVID-19 severity. Since this time, several other authors have described similar trends suggesting that there is some degree of protection from severe COVID-19 infection, especially in elderly populations^14,15^. These observations and the underlying immunomodulatory potential of BCG have prompted several worldwide clinical trials including the BADAS trial in the US (www.bcgbadas.org) to evaluate the impact of BCG vaccination on the severity and rate of COVID-19 infection.

Employing unsupervised machine learning methods with adjustment for numerous variables and potential established confounders associated with mortality, we evaluated the association between covariates designated a priori including BCG vaccination programs and mortality associated with COVID-19 at a country level utilizing pre-specified inclusion criteria.

## Methods

Countries were selected for model inclusion based on predefined criterion. Inclusion criteria included: more than 2000 cases as of May 5, 2020, population greater than 5 million, and land area greater than 1000 km^2^ (to exclude city-states with the potential for non-representative population densities). Exclusion criteria included countries where BCG program start year could not be ascertained.

All data leveraged originated from publicly available data sources (Supplementary Table 1). A set of potential disease related mortality drivers spanning seven domains—socio-economic, health system readiness, environmental, existing disease burden, demographics, vaccination programs, and response to the pandemic were selected a priori (Supplementary Table 2). COVID-19 specific mortality (CRM) was the primary outcome, defined as deaths related to COVID-19 per million population assessed 30 days after 100 reported cases.

Analysis was conducted in a stepwise manner. We sought to group countries into comparable clusters based on previously described CRM drivers. The clusters were established by using unsupervised machine learning segmentation methodology as described in more detail below. This algorithm attempts to divide the entire population (in this case countries) into groups, based on the input variables, such that similar observations (countries) get grouped together and observations (countries) which are very different go into separate clusters.

However, prior to performing this clustering of countries, the selection of distinct input variables was performed. We first assessed the correlation amongst pre-determined variables related to CRM (Supplementary Fig. 1) which demonstrated substantial correlation between several explanatory variables. Therefore, exploratory factor analysis, an unsupervised machine learning method to reduce the original set of explanatory variables, was performed. The optimum number of factors were chosen using the scree plot (Supplementary Fig. 2). From the scree plot, we observed that the decline in total within cluster SS flattened out after 7 factors with little change in TSS happening thereafter, hence the 7 factor solution was chosen (Supplementary Table 3a,b)^16^. Varimax rotation was used to maximize the loading of each variable on a single factor. From each factor group, variables were chosen as inputs for subsequent clustering and multiple regression analysis based on loading characteristics and expert consensus where loading values were similar. Given the large size of the first factor group, three variables were selected. Population density was considered as a distinct group given low loading (below 0.3) value and included in addition to one other variable from group 6. There was low variation of values for factors in group 7 thus no variables were included from this group. From the factor analysis, six variables were selected for cluster analyses including—GDP per capita, population, population density, temperature (Celsius), percentage of the population above 65 years of age, and stringency index (SI) (a measure of country level interventions in response to COVID-19)^17^.

Countries were then clustered utilizing the k-means algorithm, an unsupervised machine learning method^18^. The optimal number of clusters was determined using the average silhouette coefficient and Dunn Index (Supplementary Table 4, Supplementary Fig. 3). Countries within a cluster were then compared on the basis of categorical metrics related to BCG vaccination programs including if the country’s BCG vaccination program was active and at least 40 years old or 15 years old based on prior works indicating a reduction of vaccination efficacy after a period of 15–40 years^19,20^. Deaths per million from COVID-19 30 days after each country crossed 100 reported cases was compared for countries with currently active universal BCG vaccination programs and for either the preceding 40 or 15 years and those without such programs within a cluster. Countries within each cluster demonstrated lower coefficients of variation in testing rates compared to the whole population, and therefore normalization of testing rates was not performed.

To explore whether the findings were robust compared to alternate analytical approaches, we performed sensitivity analyses using linear regression models analyzing variables from each of the factor groups and CRM as the dependent variable. Additionally, age stratified CRM data 57 days after 100 cases (available for 7 countries for comparable periods) was analyzed for the population under 40 years compared with percent BCG coverage for the population 40 years or younger. Age less than 40 was used since the data for yearly BCG vaccine coverage for infants is reported most reliably from 1980 onwards^21^. The rate of and analytic strategy utilized for variables with missing information is presented in Supplementary Table 5. AP and AMK had full access to all the data in the study and take responsibility for the integrity of the data and the accuracy of the data analysis. The data that support the findings of this study are available from the corresponding author upon reasonable request. RStudio V 1.3.959 (Boston, MA, USA) was used for analysis.

## Results

**Figure 1:**
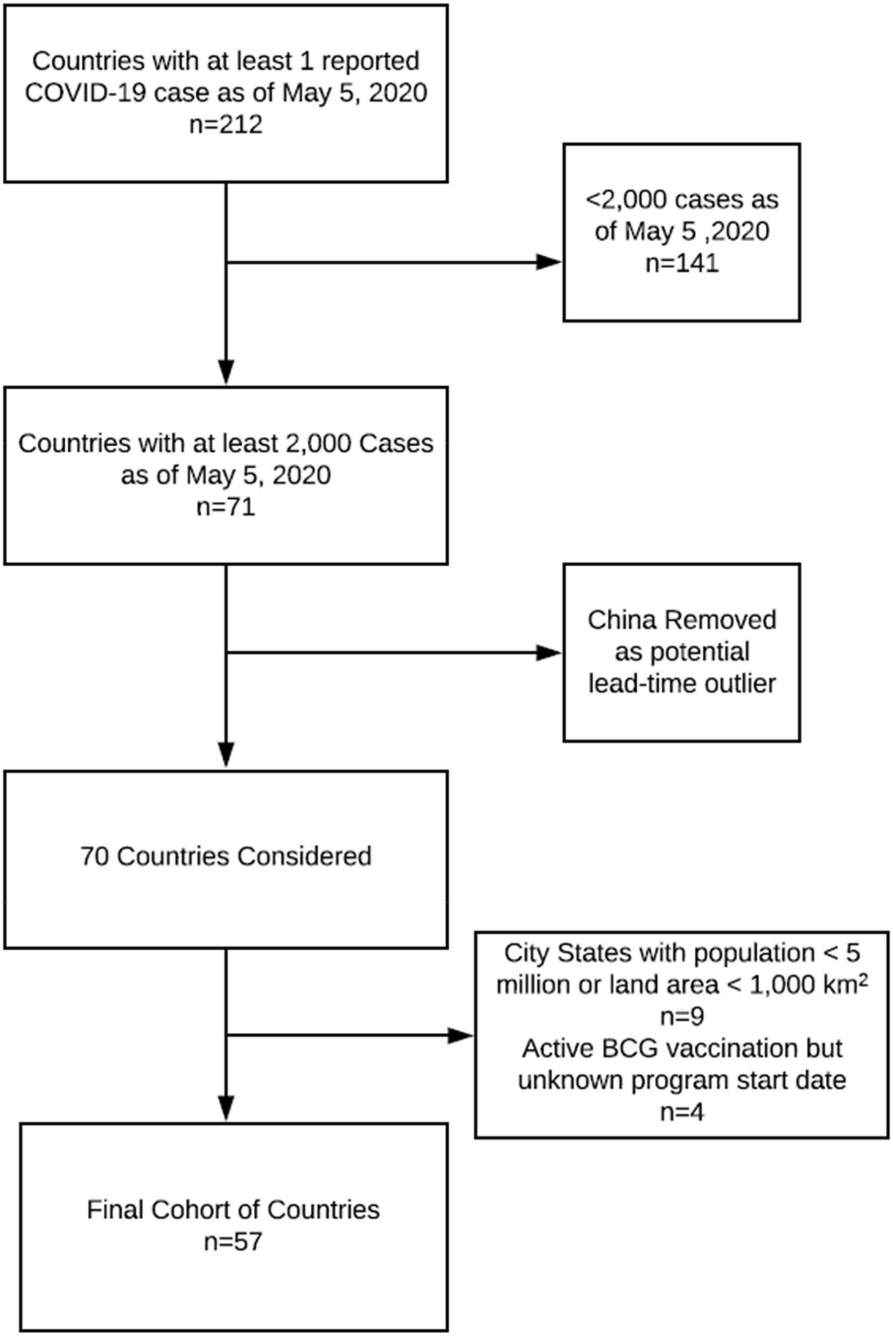
Consort diagram for the selection of countries included in evaluation. 212 countries and territories were initially screened with at least 1 case of COVID-19 as of May 5, 2020. Based on predetermined inclusion and exclusion criteria, 57 countries were included in the analysis. Figure was created using Lucidchart available at https://www.lucidchart.com/pages/.

Of 212 countries/territories, 57 countries were included in analysis (Fig. 1). Nine city states with insufficient land area or population and 141 countries with insufficient cases were excluded. Four countries met inclusion criteria but start dates for BCG vaccination programs were not available. China was excluded from the analysis as it was the first country to report widespread cases of the virus and therefore might have introduced a lead time bias or been affected by the inability to prepare as other countries were given.

Factor analysis resulted in the identification of six, distinct variables including GDP per capita, population, population density, temperature, percent population above 65 years, and stringency index (Table 1). Variables related to BCG administration were part of a distinct factor group. Countries within clusters had lower coefficient of variation for both COVID-19 testing rates and Global Health Security Agenda (GHSA) scores, compared to the overall population. Two cluster solutions, with 6 and 9 clusters, demonstrated the highest scores (Dunn Index and Silhouette Score). Since findings were similar between the 6 and 9 cluster groups and cluster 9 only included 1 country in the 9-cluster solution (Supplementary Table 6), data for the remainder of the manuscript is presented from the six-cluster solution.

**Table 1:** Simplified composition of 6 clusters included for analysis.

	Cluster 1	Cluster 2	Cluster 3	Cluster 4	Cluster 5	Cluster 6
Number of Countries	4	7	7	7	12	20
GDP for Capita^a^	3	1	2	6	4	5
% Population > 65 y/o^a^	4	2	1	6	3	5
Average Temperature^a^	3^b^	6	3^b^	1	5	2
COVID testing/million at 30 cases after first 100 cases^a^	2	1	4	6	3	5
Coefficient of Variation for COVID testing^c^	60.8	29.1	76.3	62.2	69.7	96.9
Stringency Index^a^	4^b^	4^b^	6	2^b^	1	2^b^
Overall GHSA score^a^	3	2	1	5	4	6
Coefficient of Variation for GHSA^d^	16.9	8.5	10.2	20.3	15	27.3
Countries comprising each cluster	Belgium Israel Netherlands South Korea	Austria Canada Denmark Finland Ireland Norway Switzerland	Australia France Germany Japan Sweden The United Kingdom The United States of America	Bangladesh Brazil India Indonesia Nigeria Pakistan Philippines	Czech Republic Greece Hungary Italy Kazakhstan Poland Portugal Romania Russia Serbia Spain Ukraine	Afghanistan Algeria Cameroon Chile Colombia Ecuador Egypt Ghana Iran Iraq Malaysia Mexico Morocco Oman Peru Saudi Arabia South Africa Thailand Turkey Uzbekistan

^a^Ranked 1–6, highest to lowest.

^b^Represents tie in a category.

^c^Coefficient of variation for the population Covid Testing/Million (30 days after 100 cases) was 103.5. Variation is lower in the clusters than the general population.

^d^Coefficient of variation for the population GHSA was 26.1. For all but cluster 6, there is less variation in the clusters than the population.

**Figure 2:**
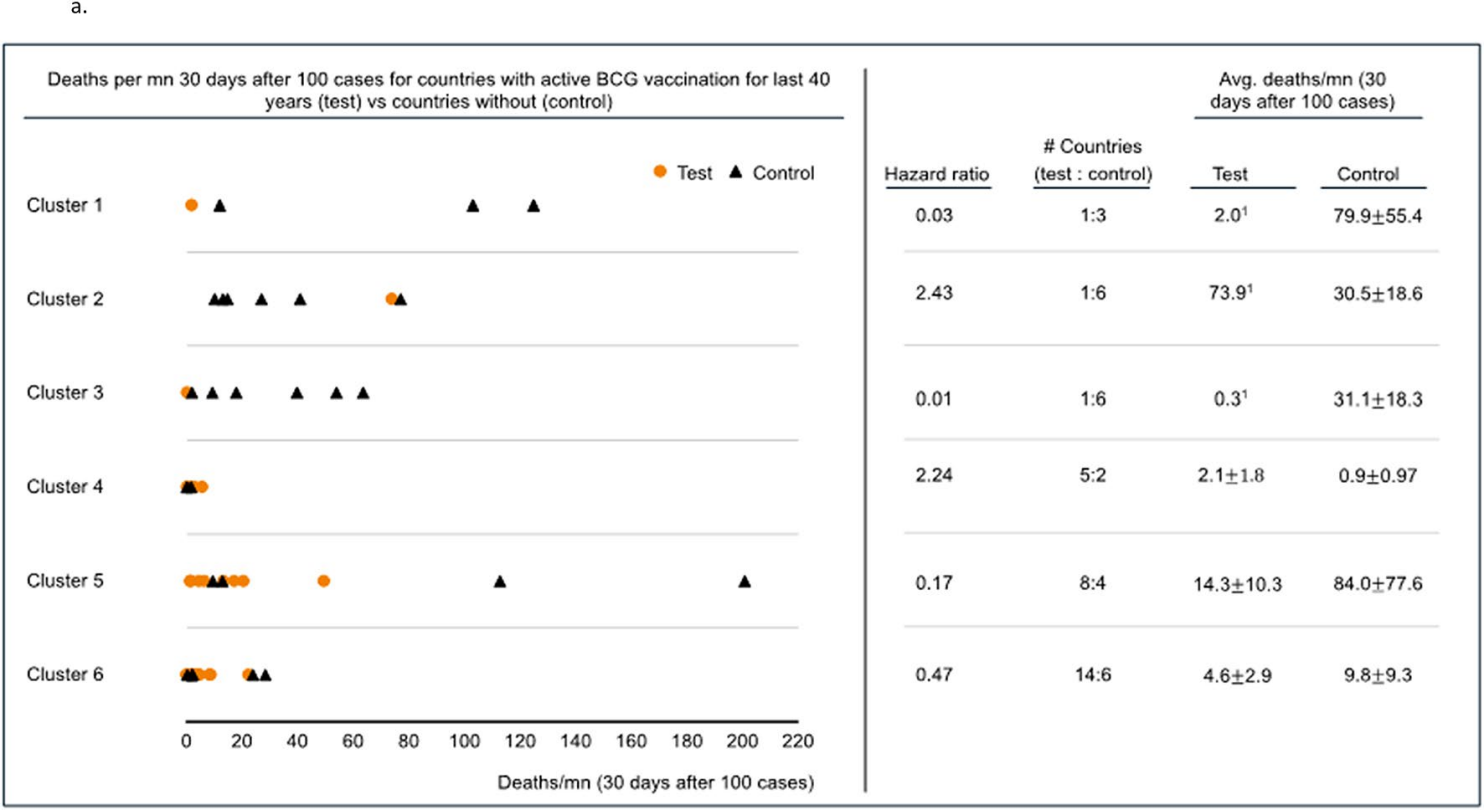

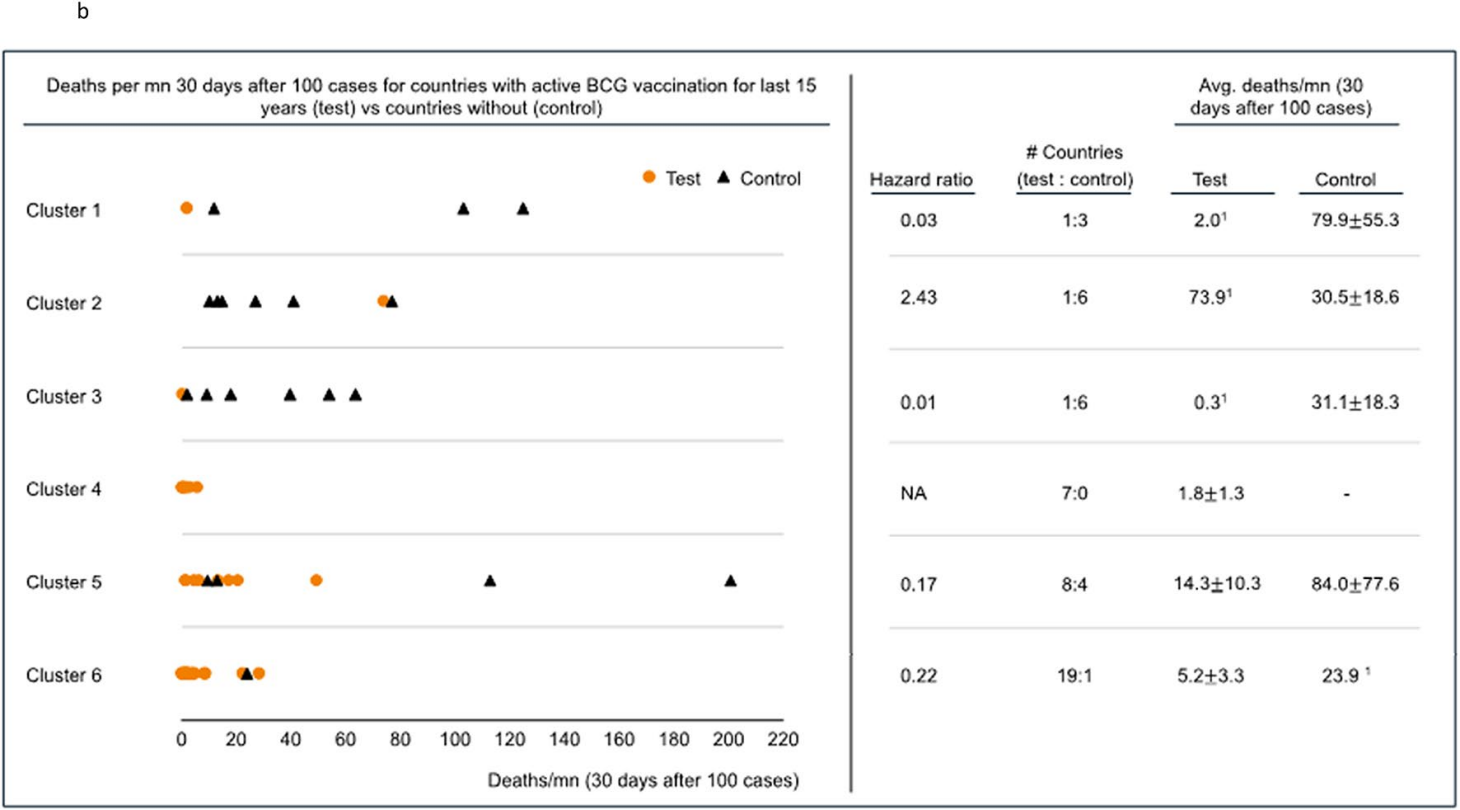
(**a**) Deaths per million 30 days after 100 cases were reached for countries with an active BCG vaccination program for the 15 years preceding the pandemic (Test) vs countries without BCG vaccination programs (Control). Cluster 4 contains no comparator and CRM was generally low. The results in cluster 2 are possibly driven by Ireland which has endured significant CRM among at risk populations. (**a**) and (**b**) were created using R Studio for windows (1.3.1093) at https://rstudio.com/; R for windows (4.0.3) at https://www.r-project.org/; and Microsoft Powerpoint Version 2008, Build 13127.20760. URL—https://www.microsoft.com/en-in/. (**b**) Deaths per million 30 days after 100 cases were reached for countries with an active BCG vaccination program for the 40 years preceding the pandemic (Test) vs countries without BCG vaccination (Control). The results in cluster 2 are possibly driven by Ireland which has endured significant CRM among at risk populations.

Deaths per million related to COVID-19 (CRM) was assessed 30 days after each included country reported 100 cases. Five of 6 clusters allowed division and comparison of CRM by the presence or absence of BCG vaccination programs for the preceding 15 years (BCG15) (Fig. 2a). The remaining cluster composed exclusively countries with BCG vaccination programs (no comparison group-cluster 2). All 6 clusters allowed division and comparison of CRM by the presence or absence of BCG vaccination programs in the preceding 40 years (BCG40) (Fig. 2b). Four of 5 clusters demonstrated lower mortality when they had BCG15 and 4 of 6 clusters demonstrated the same association with BCG40. For BCG40, specificity, clusters 1, 3, 5, and 6 demonstrated improved CRM with hazard ratios of 0.03, 0.01, 0.17, and 0.47, respectively. Cluster 2 and 4 demonstrated worse CRM with hazard ratios of 2.43 and 2.24, respectively. The results from the 9-cluster analysis were similar (Supplementary Table 7). Granular data regarding clustering is presented in Supplementary Table 8a/b.

Univariate regression analysis demonstrated that the percentage of the population above 65, total 2020 population, BCG15, average temperature, GDP per capita, Stringency Index, and BCG40 were significantly associated with CRM (Table 2). On multivariate analysis, only the presence of BCG15 (reduction of CRM by 71% (95% CI 53–89%), total population (for every 1 million person increase there was a 1% decrease in CRM (95% CI 0.53–1.47%), and share of the population above 65 years (CRM increased by 10% for each percent increase in population over 65 (95% CI 2–18%) were shown to be significantly associated with CRM. Percent coverage metrics for vaccinations including RCV1 (Rubella), MCV1 (Measles) and OPV (Polio) were forced into the model and were not significantly associated with CRM.

**Table 2:** Results of linear regression analysis.

	Univariate	Multivariate				
Variable	Estimate (95% CI) (transformed from log)	P	Direction of relationship	Estimate (95% CI) (transformed from log)^a^	P	Direction of relationship
% Population over 65 years old	1.184 ± 0.064	P < 0.001	Positive	1.1 ± 0.07	0.009	Positive
Total 2020 population	0.99 ± 0.006	P < 0.001	Negative	0.99 ± 0.0047	0.03	Negative
BCG vaccination in the preceding 15 years	0.1 ± 0.054	P < 0.001	Negative	0.29 ± 0.18	0.011	Negative
Population density	1 ± 0.003	Not significant	Not significant		Not significant	Not significant
Average temperature	0.913 ± 0.04	P < 0.001	Negative		Not significant	Not significant
GDP per capita	1 ± 0	P < 0.001	Positive		Not significant	Not significant
Stringency Index	0.97 ± 0.022	0.012	Negative		Not significant	Not significant
BCG vaccination in the preceding 40 years	0.235 ± 0.136	0.001	Negative		Not significant	Not significant
BCG coverage in last 40 years	0.988 ± 0.008	0.007	Negative		Not significant	Not significant
Polio vaccine coverage	1.009 ± 0.012	Not significant	Not significant		Not significant	Not significant
Polio vaccine duration	1.009 ± 0.013	Not significant	Not significant		Not significant	Not significant
Measles vaccine coverage	0.005 ± 0.008	Not significant	Not significant		Not significant	Not significant
Measles vaccine duration	0.0004 ± 0.0006	Not significant	Not significant		Not significant	Not significant

The percentage of the population over 65 years of age was associated with higher rates of CRM such that for every percent increase in population over 65 years old, CRM increased by 10% (95% confidence interval 2–18%). The total population of a country was associated with improved CRM (for every unit increase there was a 1% decrease in CRM (95% CI 0.53–1.47%). An active BCG vaccination program for the preceding 15 years was shown to reduce CRM significantly by 71% (95% CI 53–89%). A negative relationship suggests that the variable results in a decrease in CRM and vice versa.

^a^The antilog of all estimates. For example, when % population above 65 yrs. increases by 1%, the deaths/mn on an average increase by 1.10 times, i.e., 10% (with a 95% CI of 1.02–1.18 or 2–18%). Likewise, when a country has BCG coverage in the last 15 years, the deaths/mn decreases by 0.29 times, i.e., 71% (with a 95% CI of 0.11–0.47 or 89–53%).

Age stratified CRM for those under 40 years of age in relation to BCG coverage percentage for the same population was compared for 7 countries where the latest data was available (Table 3). Countries with no or low coverage for BCG vaccination in the population under 40, including the population between 30–39 and 20–29, had higher CRM for the same age groups, with the exception of Switzerland which had no reported COVID-19 related deaths in the 20–29 year age group.

**Table 3:** Analysis of CRM for populations aged < 40 compared with the percent BCG vaccination rate in the same age group.

Countries	CRM for population< 40 years per million population< 40 years	BCG Coverage % under 40 years	CRM for population 20–29 years per million population 20–29 years	BCG Coverage % for age groups of 20–29	CRM for population 30–39 per million population 30–39 years	BCG Coverage % for age groups of 30–39
Sweden	3.4	0	4.6	0	8.4	0
Italy	2.3	0	1.2	0	6.3	0
Netherlands	1.8	0	2.4	0	4.7	0
Switzerland	1.2	Not reported	0.0	0	4.3	Not reported
Czech Republic	1.1	74	0.8	78	2.6	98
Poland	0.7	94	0.0	96	2.3	95
South Korea	0.0	77	0.0	84	0.1	53

Countries with lower rates of BCG coverage generally have higher rates of CRM suggesting that, even in a less vulnerable population, BCG vaccination is associated with improved CRM.

## Discussion

Using strict criteria designated a priori we have demonstrated an independent association between BCG vaccine administration programs active for the preceding 15 years and reduced CRM (71% reduction, OR 0.29 ± 0.18). BCG15 was more strongly associated than BCG40 with CRM suggesting, as would be expected, improved efficacy for more recently administered vaccinations. It might also represent improved data reliability or vaccination administration for more recent programs. CRM was higher for populations over 65 years of age. CRM was lower for countries with higher total population which might suggest that transmission dynamics differ, testing rates are lower, that they are more able to mount a response, or an as yet identified factor is present^22^. OPV, MCV1, and RCV1 vaccination status was not associated with decreased CRM suggesting that it is not the global presence of vaccination associated with CRM but specifically BCG vaccination. Finally, BCG vaccination was associated with decreased CRM even in the population under 40 years of age.

Because no two countries are identical, but several countries may be considered similar, we clustered countries together based on factors determined to be independent from each other using machine learning in order to refine previous methods of comparing countries without such similarities. The variance between COVID-19 testing for countries in each cluster was lower than that of the population suggesting that clusters which were homogenous within themselves and distinct from other clusters. Several potential mechanisms may explain the variability of the association between BCG and CRM between clusters.

Since we first described the association between BCG vaccination policies and CFR, several additional studies have corroborated this finding^13,23^. Sala et al. demonstrated that TB infection and BCG vaccination strategies were associated with decreased incidence and mortality related to COVID-19^15^. Shet et al. demonstrated a 5.8-fold decrease in COVID-19 related mortality for populations with BCG vaccination^24^. These studies, including our prior work, are hampered by the quality of the data from which they derive their analysis as well as by the inability to adequately include and capture all potential confounding variables. The present analysis is strengthened by the comprehensive nature of the analysis not present in prior works as well as the a priori definition of input and outcome variables. The clinical validity of increased CRM for populations older than age 65^25^ has been well demonstrated. That this association was also determined in the machine learning models further strengthens the finding that BCG was associated with lower CRM.

Efficient contact tracing, isolation, and rapid testing, as part of a larger program of countermeasures, have proven effective at controlling SARS-CoV-2 outbreaks in areas such as China and South Korea. Neither the implementation of rapid contract tracing with targeted isolation, widespread testing, nor regional lockdowns had been as readily deployed in many countries upon the analysis CRM in the present work^26,27^. Hensel et al. found that for countries with high testing rates, BCG vaccination no longer correlated with incidence^28^. However, in countries with current BCG vaccination policies and higher rates of testing, BCG vaccination remained significantly associated with reduced rates of CRM^28^. For Israeli adults aged 35–41 with symptoms suggestive of COVID-19, no difference was found in incidence for those born during BCG vaccination programs or those born just after they ended. This represented a young, 6000-person cohort with only 2 cases of severe disease^29^ but did highlight the need for data quality and completeness. Our work is further strengthened by evaluating COVID-19 mortality in 7 countries with complete vaccination data for the population under 40 where BCG vaccination continued to demonstrate an association with improved CRM.

More recently, two conflicting reports have also been published. Escobar et al.^30^ performed an analysis of several factors associated with reducing CRM via BCG vaccination program. Overall, BCG vaccination programs and program coverage were related to decreased CRM especially in the early pandemic prior to more organized responses. Similarly, they observed that CRM was greater in countries with older populations. This consistency with our current work would suggest that there is a relationship between CRM and BCG vaccination status. However, recently, Wassenaar et al.^31^ evaluated the CRM in 18 countries that had introduced the BCG vaccine in the 1950s, specifically to evaluate the effect of BCG in the population over 65 years of age. They did not find that BCG vaccination policy was related to CRM over a similar time period to the current manuscript. However, in the work Wassenaar et al., the restriction of the analysis to only 18 countries without the stringent selection criteria and clustering performed in the current study is a major limitation of these findings.

The magnitude of the association between BCG and CRM must be taken in context with local responses to COVID-19. For example, in cluster 1, only South Korea (SK) had an active BCG vaccination program and the rates of CRM were lower in this cohort. This effect was again demonstrated for people in SK under the age of 40. The lower rates of CRM in SK might represent BCG vaccination, the efforts of the public health department, or an unknown/unmeasured variable^32^. Similarly, in cluster 2, Ireland was the only country with an active BCG vaccination program, though with decreases in vaccination rates starting in 2005, but with higher levels of CRM which might more closely represent delay in taking COVID-19 measures^33^. In spite of such country specific possibilities, the general association of BCG vaccination status consistently demonstrated improved CRM. Finally, this work was performed near the beginning of the pandemic before a better understanding of pandemic mitigation and the critical care of COVID-19 patients had been achieved. We therefore hypothesize that impact of innate protection by BCG vaccination would be better detected during this timepoint than later in the pandemic when a better understanding of critical care has decreased the mortality rate.

We interpret our own findings with a cautionary note since there are numerous potential measured and unmeasured confounding variables including rates of BCG vaccination compliance, age at vaccination, potential strain differences among BCG vaccines, as well as regional variations within countries, a lack of a verified metric to measure country-level COVID-19 response effectiveness, no measures of health system capacity to provide effective, critical care, and other, as yet identified factors. The rates of adequate and accurate CRM reporting may vary by country and vary more widely in certain countries with higher BCG vaccination rates owing to the robustness of the public health infrastructure thus increasing the apparent effect of BCG. We agree with the sentiments of the World Health Organization and caution against routine BCG vaccination for the prevention of COVID-19 until prospective trials are completed. It is unclear if the protection from neonatal vaccination with BCG is transferrable to those receiving vaccination as an adult and how long such protection lasts. That is why some of the authors have initiated NCT04348370 (BADAS) trial in the US, joining other trials evaluating BCG administration for either COVID-19 prevention or disease severity reduction including: national clinical trial (NCT)04348370 (BADAS, USA), NCT04327206 (BRACE, Australia), NCT04328441 (BCG-CORONA, Netherlands), and NCT04350931 (Egypt).

This analysis represents an attempt to utilize machine learning methods to address important questions in the field of medicine during a global health emergency which might foster accelerated research in medicine and epidemiology.

## Conclusion

For countries included in our analysis using an a priori, rigid entry criteria, the presence of an active BCG immunization program for the past 15 years and total population are associated with improved COVID-19 specific mortality while the share of the population over 65 years of age is associated with increased CRM. For the included countries BCG15 vaccination programs are associated with a 71% reduction in the risk for CRM independent of population, population density, temperature, share of population above 65 years, and the stringency index of each country. A reduction in CRM was observed in 80% of country clusters for BCG15. This ecological analysis provides the most robust data regarding the association of COVID-19 specific mortality and BCG vaccination programs. These findings suggest that BCG vaccination is one of many potential additions to our armamentarium in the fight to reduce mortality related to COVID-19.

## Supplementary information

Supplementary Information.
