# Supplementary material for: The association of Coronavirus Disease-19 mortality and prior bacille Calmette-Guerin vaccination: a robust ecological analysis using unsupervised machine learning
Source: Sci Rep. 2021 Jan 12;11:774. doi: 10.1038/s41598-020-80787-z (PMC7804196; doi:10.1038/s41598-020-80787-z)

Supplementary table The association of Coronavirus Disease-19 mortality and prior bacille Calmette-Guerin vaccination: a robust ecological evaluation using unsupervised machine learning

Nathan A. Brooks MD^1*^, Ankur Puri MBA, MTech^2^*, Sanya Garg MSc^2^, Swapnika Nag MBA^2^ Jacomo Corbo PhD^4^, Anas El Turabi MD, PhD^5^, Noshir Kaka MMS^6^, Rodney W. Zemmel PhD^7^, Paul K. Hegarty MD^8**^ and Ashish M. Kamat MD, MBBS^1**^

* These authors contributed equally to this work

**These authors share senior authorship

1. The University of Texas MD Anderson Cancer Center, Department of Urology, Houston, TX, United States

2. McKinsey & Company, Gurgaon, Haryana, India

3. McKinsey & Company, Bengaluru, Karnataka, India

4. QuantumBlack a McKinsey Company, London, UK

5. McKinsey & Company, Waltham, MA, United States

6. McKinsey & Company, Mumbai, Maharashtra, India

7. McKinsey & Company, New York City, NY, United States

8. Mater Private Hospital, Department of Urology, Cork, Ireland

Corresponding Author:

Ashish M. Kamat, MD

Department of Urology

University of Texas MD Anderson Cancer Center

Houston, TX, USA

Tel: +1 713-792-2121

Electronic address: [akamat@mdanderson.org](mailto:akamat@mdanderson.org)

Supplementary Figures: 3

Supplementary Tables: 8

Running Title: Association of COVID-19 severity and BCG vaccination

Supplementary Table 1: Variables were assigned a priori and abstracted from each source with secondary sources utilized as needed. The most recent date of data access is listed in the table. Data was accessed from each source between April 15, 2020 to May 1, 2020

| **Factor** | **Variable** | **Source Link** | **Source Name** | **Supplementary source** |
| --- | --- | --- | --- | --- |
| **Economic Factors** | GDP per capita | <https://en.wikipedia.org/wiki/List_of_countries_by_GDP_(nominal)_per_capita#cite_note-5> | World Bank |  |
| **Demographic Factors** | Population | <https://www.worldometers.info/world-population/> | Worldometer |  |
|  | Population Density | <https://data.worldbank.org/indicator/EN.POP.DNST> | World Development Indicators |  |
|  | % pop above 65 yrs. | <https://www.indexmundi.com/factbook/countries> | Index Mundi |  |
|  | % pop above 80 yrs. | <https://data.worldbank.org/indicator/SP.POP.80UP.FE.5Y> | World Bank database |  |
|  |  | <https://data.worldbank.org/indicator/SP.POP.80UP.MA.5Y> |  |  |
|  | Average Household Size | <https://en.wikipedia.org/wiki/List_of_countries_by_number_of_households> | Wikipedia |  |
| **Geographic Factors** | Average Temperature (Feb, Mar and Apr) | <https://en.wikipedia.org/wiki/List_of_cities_by_average_temperature> | Wikipedia |  |
| **COVID related factors** | Tests/mn (30 days after 100 cases) | <https://ourworldindata.org/coronavirus-testing>  *(In case testing data for exact data was unavailable, data was taken for the closest available date)* | Our World In Data | <https://www.worldometers.info/coronavirus/> |
|  | Deaths per mn (30 days after 100 cases) | <https://data.humdata.org/dataset/novel-coronavirus-2019-ncov-cases> | John Hopkins | <https://www.worldometers.info/coronavirus/> |
| **BCG Immunization** | # Countries with BCG immunization at any point in time | <http://www.bcgatlas.org/> | BCG atlas | Vaccine academic journals for respective countries |
|  | # Countries with current BCG Immunization | <http://www.bcgatlas.org/> | BCG atlas | Vaccine academic journals for respective countries |
|  | Population above 65 yrs. Immunized or not | <http://www.bcgatlas.org/> | BCG atlas | Vaccine academic journals for respective countries |
|  | Ongoing BCG program since 70 years | <http://www.bcgatlas.org/> | BCG atlas | Vaccine academic journals for respective countries |
|  | Years since start of BCG policy | <http://www.bcgatlas.org/> | BCG atlas | Vaccine academic journals for respective countries |
|  | # Countries with coverage data | <https://apps.who.int/immunization_monitoring/globalsummary/timeseries/tswucoveragebcg.html> | WHO |  |
|  | Average BCG coverage in population | <https://apps.who.int/immunization_monitoring/globalsummary/timeseries/tswucoveragebcg.html> | WHO | UN age structure population % estimates |
| **Rubella coverage** | RCV1 Coverage | <https://apps.who.int/immunization_monitoring/globalsummary/timeseries/tswucoveragebcg.html> | WHO | UN age structure population % estimates |
|  | RCV1_Duration | <https://apps.who.int/immunization_monitoring/globalsummary/timeseries/tswucoveragebcg.html> | WHO | Vaccine academic journals for respective countries |
| **Measles Coverage** | MCV1 Coverage | <https://apps.who.int/immunization_monitoring/globalsummary/timeseries/tswucoveragebcg.html> | WHO | UN age structure population % estimates |
|  | MCV1 Duration | <https://apps.who.int/immunization_monitoring/globalsummary/timeseries/tswucoveragebcg.html> | WHO | Vaccine academic journals for respective countries |
| **Polio Vaccine** | Pol3 | <https://apps.who.int/immunization_monitoring/globalsummary/timeseries/tswucoveragebcg.html> | WHO | UN age structure population % estimates |
|  | Pol3 Duration | <https://apps.who.int/immunization_monitoring/globalsummary/timeseries/tswucoveragebcg.html> | WHO | Vaccine academic journals for respective countries |
| **Disease Burden** | % Deaths – Diabetes | <https://www.who.int/healthinfo/mortality_data/en/> -> Select indicator as “endocrine, nutritional and diabolical diseases” -> select “No. of deaths, diabetes mellitus, both sexes” -> select country and latest year  **Value calculated as :** (No. of deaths, diabetes mellitus, both sexes for country) / Population of country | WHO |  |
|  | % Deaths – Hypertension | <https://www.who.int/healthinfo/mortality_data/en/> -> Select indicator as “diseases of the circulatory system” -> select “No. of deaths, hypertensive diseases, both sexes” -> select country and latest year  **Value calculated as :** (No. of deaths, hypertensive diseases, both sexes for country) / Population of country | WHO |  |
|  | % Deaths – Cerebrovascular | <https://www.who.int/healthinfo/mortality_data/en/> -> Select indicator as “diseases of the circulatory system” -> select “No. of deaths, cerebrovascular diseases, both sexes” -> select country and latest year  **Value calculated as :** (No. of deaths, cerebrovascular diseases, both sexes for country) / Population of country | WHO |  |
|  | % Deaths - Pneumonia | <https://www.who.int/healthinfo/mortality_data/en/> -> Select indicator as “diseases of the respiratory system” -> select “No. of deaths, pneumonia, both sexes” -> select country and latest year  **Value calculated as :** (No. of deaths, cerebrovascular diseases, both sexes for country) / Population of country | WHO |  |
|  | % Deaths - Lower respiratory disease | <https://www.who.int/healthinfo/mortality_data/en/> -> Select indicator as “diseases of the respiratory system” -> select “No. of deaths, chronic lower respiratory diseases, both sexes” -> select country and latest year  **Value calculated as :** (No. of deaths, chronic lower respiratory diseases, both sexes for country) / Population of country | WHO |  |
|  | % Deaths – TB | <https://www.who.int/healthinfo/mortality_data/en/> -> Select indicator as “certain infectious and parasitic diseases” -> select “No. of deaths, tuberculosis, both sexes” -> select country and latest year  **Value calculated as :** (No. of deaths, tuberculosis, both sexes for country) / Population of country | WHO |  |
|  | % Deaths – Obesity | <https://ourworldindata.org/obesity> | WHO |  |
|  | % Deaths – HIV | <https://www.who.int/healthinfo/mortality_data/en/> -> Select indicator as “certain infectious and parasitic diseases” -> select “No. of deaths, HIV disease, both sexes” -> select country and latest year  **Value calculated as :** (No. of deaths, HIV disease, both sexes for country) / Population of country | WHO |  |
| **Government measures** | Stringency Index (12 days after 100 cases, pulled as of 30th April) | <https://www.bsg.ox.ac.uk/research/research-projects/coronavirus-government-response-tracker> | Oxford |  |
| **Efficiency of public health systems** | Overall GHSA score | [https://www.ghsindex.org/wp-content/uploads/2019/10/2019-Global-Health-Security-Index.pdf](https://en.wikipedia.org/wiki/List_of_cities_by_average_temperature) | 2019 Global Health Security Index |  |
|  | Prevention of emergence or release of pathogens | [https://www.ghsindex.org/wp-content/uploads/2019/10/2019-Global-Health-Security-Index.pdf](https://en.wikipedia.org/wiki/List_of_cities_by_average_temperature) | 2019 Global Health Security Index |  |
|  | Early detection & reporting for epidemics of potential international concern | [https://www.ghsindex.org/wp-content/uploads/2019/10/2019-Global-Health-Security-Index.pdf](https://en.wikipedia.org/wiki/List_of_cities_by_average_temperature) | 2019 Global Health Security Index |  |
|  | Rapid response to and mitigation of the spread of an epidemic | [https://www.ghsindex.org/wp-content/uploads/2019/10/2019-Global-Health-Security-Index.pdf](https://en.wikipedia.org/wiki/List_of_cities_by_average_temperature) | 2019 Global Health Security Index |  |
|  | Sufficient and robust health system To treat the sick & protect health workers | [https://www.ghsindex.org/wp-content/uploads/2019/10/2019-Global-Health-Security-Index.pdf](https://en.wikipedia.org/wiki/List_of_cities_by_average_temperature) | 2019 Global Health Security Index |  |
|  | Commitments to improving national capacity, financing, and adherence to norms | [https://www.ghsindex.org/wp-content/uploads/2019/10/2019-Global-Health-Security-Index.pdf](https://en.wikipedia.org/wiki/List_of_cities_by_average_temperature) | 2019 Global Health Security Index |  |
|  | Risk environment and vulnerability to biological threats | [https://www.ghsindex.org/wp-content/uploads/2019/10/2019-Global-Health-Security-Index.pdf](https://en.wikipedia.org/wiki/List_of_cities_by_average_temperature) | 2019 Global Health Security Index |  |
| Age stratified data | Country wise age stratified mortality 57 days after 100 cases | <https://www.statista.com/> | Statista |  |

GDP: Gross domestic product, MN: million, RCV1: Rubella vaccination, MCV1: measles vaccination, POL3: polio vaccination status, UN: United Nations, GHSA: Global Health Security Agenda

Supplementary Table 2: Variables selected a priori to represent several potential domains impacting COVID-19 related response and mortality. Broadly, they are grouped into categories addressing economic factors, demographic factors, geographic factors, disease testing, Immunizations (including BCG, Rubella, Measles, and Polio), underlying population comorbid conditions, the stringency of response, and the efficiency of public health systems.

| **Factor** | **Variable** | **Description** | **Year** |
| --- | --- | --- | --- |
| **Economic Factors** | GDP_Per_Capita | Nominal GDP per capita ($) | 2018 |
| **Demographic Factors** | Population_2020 | Population in million | 2020 |
|  | Poputltion Density | People per square km of land | 2018 |
|  | Percent_Pop_Above_65 | Distribution of population above 65 years | 2018 |
|  | Percent_Pop_Above_80 | Distribution of population above 80 years | 2018 |
|  | Household_Size_2019 | Average household size in a country | Latest year available |
| **Geographic Factors** | Avg_Temp | Temperature in Celsius averaged out for February, March, & April for cities within countries | Latest year available |
| **COVID-19 related** | Tests_Mn_30_Days_After_100th_Case | Number of tests conducted per million of population 30 days after the date of 100 cases | 2020 |
|  | Deaths_Mn_30_Days_After_100th_Case | Number of deaths due to COVID-19 per million of population 30 days after the date of 100 cases | 2020 |
| **BCG Immunization** | BCG_Immunization_Ever | Countries with active universal BCG immunisation policy at any point in time in history | 2020 |
|  | BCG_Immunization_Current | Countries with currently national active universal BCG immunisation policy | 2020 |
|  | BCG_Last_15_Coverage_Yes | Active national universal BCG immunisation policy currently and for last 15 years (uptil 2005) | 2020 |
|  | BCG_Last_40_Coverage_Yes | Active national BCG universal immunisation policy currently and for last 40 years (uptil 1980) | 2020 |
|  | BCG_Years_Of_Immunization | Years since national universal BCG immunisation policy was introduced | 2020 |
|  | BCG_Coverage_Percentage | Implementation of BCG vaccine overlayed by population age structure and years of implementation to obtain average BCG vaccine coverage in population | 2020 |
|  | Perc_BCG_Coverage_GT50 | Is BCG coverage % greater than 50% (Boolean) | 2020 |
|  | BCG_coverage_last_40yrs | Implementation of BCG vaccine overlayed by population age structure and years of implementation to obtain average BCG vaccine coverage in population - for population under 40 years | 2020 |
| **Rubella vaccine Coverage** | RCV1_Coverage | Implementation of RCV1 vaccine overlayed by population age structure and years of implementation to obtain average RCV1 vaccine coverage in population | 2020 |
|  | RCV1_Duration | Years since RCV1 immunisation policy was introduced |  |
| **Measles vaccine coverage** | MCV1_Coverage | Implementation of MCV1 vaccine overlayed by population age structure and years of implementation to obtain average MCV1 vaccine coverage in population | 2020 |
|  | MCV1_Duration | Years since MCV1 immunisation policy was introduced |  |
| **Polio vaccine coverage** | Polio_Coverage | Implementation of POL3 vaccine overlayed by population age structure and years of implementation to obtain average POL3 vaccine coverage in population | 2020 |
|  | Polio_Duration | Years since POL3 immunisation policy was introduced |  |
| **Disease Burden** | Perc_Deaths_Diabetes | % of deaths in a country due to diabetes | Latest year available |
|  | Perc_Deaths_Hypertension | % of deaths in a country due to hypertension | Latest year available |
|  | Perc_Deaths_Cerebrovascular | % of deaths in a country due to cerebrovascular disease | Latest year available |
|  | Perc_Deaths_Pneumonia | % of deaths in a country due to pneumonia | Latest year available |
|  | Perc_Deaths_Lower_Resp_Disease | % of deaths in a country due to low respiratory diseases | Latest year available |
|  | Perc_Deaths_TB | % of deaths in a country due to TB | Latest year available |
|  | Perc_Deaths_Obesity | % of deaths in a country due to Obesity | Latest year available |
|  | Perc_Deaths_HIV | % of deaths in a country due to HIV | Latest year available |
| **Government measures** | Stringency_Index | Scoring of the stringency of measures taken by the government on containment, closures, restrictions, income support, and health system policies 12 days after date of 100 cases | 2020 |
| **Efficiency of public health systems** | Overall_GHSA_Score | Overall GHSA score | 2019 |
|  | GHSA1_Emergence_Of_Disease | Prevention of emergence or release of pathogens | 2019 |
|  | GHSA2_Detection | Early detection & reporting for epidemics of potential international concern | 2019 |
|  | GHSA3_Response | Rapid response to and mitigation of the spread of an epidemic | 2019 |
|  | GHSA4_Sufficient_System | Sufficient and robust health system To treat the sick & protect health workers | 2019 |
|  | GHSA5_Commitments | Commitments to improving national capacity, financing, and adherence to norms | 2019 |
|  | GHSA6_Risk | Risk environment and vulnerability to biological threats | 2019 |

Supplementary Table 3:

a. Summary of 7 factor rotation evaluation for factor loading. Proportion variance defines the variation captured by the factor where 0 is not captured and 1 is perfect capture. Cumulative variance shows total variance captured by the number of factors considered. Values greater than 0.6 indicate excellent capture. Secene factor inclusion was determined to be optimal.

| **Summary table for 7 factor rotation** | Factor 1 | Factor 2 | Factor 3 | Factor 4 | Factor 5 | Factor 6 | Factor 7 |
| --- | --- | --- | --- | --- | --- | --- | --- |
| Sum of Squares loading | 9.32 | 4.43 | 3.67 | 2.41 | 2.18 | 2.05 | 1.98 |
| Proportion Variance | 0.25 | 0.12 | 0.1 | 0.06 | 0.06 | 0.05 | 0.05 |
| Cumulative Variance | 0.25 | 0.36 | 0.46 | 0.52 | 0.58 | 0.63 | 0.69 |
| Proportion Explained | 0.36 | 0.17 | 0.14 | 0.09 | 0.08 | 0.08 | 0.08 |
| Cumulative Proportion | 0.36 | 0.53 | 0.67 | 0.76 | 0.85 | 0.92 | 1 |

b. Summary of factors which were included into the seven-factor grouping. MR – measure factor loading which is similar to the correlation coefficient between a variable and the factor (MR 1 indicates measure of correlation between variable and factor 1), h2 – measure of communalities i.e proportion of each variable's variance that can be explained by the factors, u2 – measure of unique variance i.e proportion of a variable's variance that cannot be explained by the factors, com – measure of factor complexity i.e how many variables load significantly onto two or more factors. Max factor loading indicates the factor group that the variable best belongs with. If max factor loading is greater than 0.3 then the variable is relevant for the factor

| **Factor Assignment** | **Feature** | **MR1** | **MR2** | **MR3** | **MR5** | **MR4** | **MR7** | **MR6** | **h2** | **u2** | **com** | **Max Factor Loading** |
| --- | --- | --- | --- | --- | --- | --- | --- | --- | --- | --- | --- | --- |
| 1 | Overall_GHSA_Score | 0.97 | -0.18 | 0.05 | 0.03 | 0.13 | 0.1 | 0.04 | 1 | -0.0018 | 1.1 | 0.97 |
| 1 | GHSA4_Sufficient_System | 0.9 | -0.19 | 0.06 | -0.1 | 0.1 | 0.08 | 0.02 | 0.88 | 0.1206 | 1.2 | 0.9 |
| 1 | GHSA3_Response | 0.86 | -0.08 | -0.03 | 0.01 | 0.12 | 0.16 | 0.2 | 0.82 | 0.1798 | 1.2 | 0.86 |
| 1 | GHSA1_Emergence_Of_Disease | 0.85 | -0.19 | 0.21 | 0.14 | 0.07 | 0.08 | -0.09 | 0.84 | 0.1577 | 1.4 | 0.85 |
| 1 | GHSA6_Risk | 0.82 | -0.14 | 0.19 | 0.14 | 0.13 | -0.13 | -0.25 | 0.84 | 0.1591 | 1.6 | 0.82 |
| 1 | RCV1_Duration | 0.8 | -0.19 | 0.19 | 0.02 | 0.09 | -0.22 | -0.34 | 0.88 | 0.1225 | 1.8 | 0.8 |
| 1 | GHSA2_Detection | 0.74 | -0.29 | -0.08 | 0.01 | 0.26 | 0.12 | 0.09 | 0.73 | 0.2719 | 1.7 | 0.74 |
| 1 | GDP_Per_Capita | 0.73 | -0.31 | 0.14 | 0.16 | -0.02 | -0.24 | -0.25 | 0.79 | 0.2125 | 2.1 | 0.73 |
| 1 | GHSA5_Commitments | 0.69 | -0.01 | 0.06 | 0.06 | -0.11 | 0.02 | 0.09 | 0.5 | 0.5008 | 1.1 | 0.69 |
| 1 | Percent_Pop_Above_65 | 0.68 | -0.16 | 0.65 | 0.1 | 0.11 | -0.12 | -0.04 | 0.94 | 0.0624 | 2.3 | 0.68 |
| 1 | RCV1_Coverage | 0.67 | -0.16 | -0.03 | -0.04 | 0.1 | -0.18 | -0.47 | 0.74 | 0.258 | 2.2 | 0.67 |
| 1 | Stringency_Index | -0.65 | 0.1 | -0.04 | -0.21 | -0.07 | 0.17 | -0.2 | 0.55 | 0.4511 | 1.6 | 0.65 |
| 1 | Percent_Pop_Above_80 | 0.62 | -0.18 | 0.61 | 0.12 | 0.11 | -0.11 | -0.03 | 0.82 | 0.1755 | 2.4 | 0.62 |
| 2 | BCG_Years_Of_Immunization | -0.03 | 0.93 | 0.09 | -0.06 | -0.04 | -0.06 | -0.09 | 0.9 | 0.103 | 1.1 | 0.93 |
| 2 | BCG_Last_40_Coverage_Yes | -0.24 | 0.68 | 0 | -0.21 | -0.01 | 0.37 | 0.07 | 0.7 | 0.2994 | 2.1 | 0.68 |
| 2 | BCG_coverage_last_40yrs | -0.16 | 0.68 | -0.33 | 0.23 | -0.12 | 0.21 | -0.21 | 0.75 | 0.2464 | 2.4 | 0.68 |
| 2 | Perc_BCG_Coverage_GT50 | -0.18 | 0.67 | -0.2 | 0.07 | 0.02 | 0.27 | -0.4 | 0.76 | 0.2426 | 2.5 | 0.67 |
| 2 | BCG_Last_15_Coverage_Yes | -0.52 | 0.66 | -0.18 | -0.19 | -0.05 | 0.25 | 0.18 | 0.87 | 0.125 | 2.8 | 0.66 |
| 2 | BCG_Coverage_Percentage | -0.09 | 0.59 | -0.32 | 0.31 | -0.12 | 0.16 | -0.34 | 0.71 | 0.2857 | 3.2 | 0.59 |
| 2 | BCG_Immunization_Current | -0.55 | 0.59 | -0.12 | -0.21 | -0.02 | 0.25 | 0.19 | 0.81 | 0.1884 | 3 | 0.59 |
| 2 | BCG_Immunization_Ever | -0.16 | 0.53 | 0.09 | -0.04 | -0.07 | -0.06 | -0.01 | 0.33 | 0.6736 | 1.3 | 0.53 |
| 2 | Perc_Deaths_Influenza | 0.17 | -0.35 | 0.04 | 0.05 | -0.14 | 0.02 | -0.13 | 0.19 | 0.8072 | 2.2 | 0.35 |
| 3 | Perc_Deaths_Cerebrovascular | 0.11 | 0.07 | 0.82 | -0.11 | 0.17 | 0.3 | -0.18 | 0.85 | 0.1537 | 1.6 | 0.82 |
| 3 | Perc_Deaths_Obesity | -0.23 | 0.15 | 0.74 | -0.06 | 0.1 | -0.01 | 0.09 | 0.64 | 0.361 | 1.4 | 0.74 |
| 3 | Avg_Temp | -0.35 | 0.02 | -0.58 | -0.16 | 0.11 | 0.27 | 0.23 | 0.63 | 0.3681 | 2.8 | 0.58 |
| 3 | Perc_Deaths_Lower_Resp_Disease | 0.2 | -0.21 | 0.58 | 0.06 | -0.17 | -0.04 | 0.04 | 0.46 | 0.5438 | 1.8 | 0.58 |
| 3 | Household_Size_2019 | -0.48 | 0.24 | -0.52 | -0.26 | -0.02 | 0.12 | 0.07 | 0.65 | 0.3544 | 3.1 | 0.52 |
| 4 | MCV1_Duration | 0.24 | -0.05 | 0.07 | 0.06 | 0.96 | 0 | 0.07 | 0.99 | 0.0096 | 1.2 | 0.96 |
| 4 | MCV1_Coverage | 0.22 | -0.04 | 0.04 | 0.06 | 0.95 | 0 | 0.03 | 0.96 | 0.0401 | 1.1 | 0.95 |
| 5 | Polio_Coverage | 0.13 | -0.06 | 0.04 | 0.95 | 0.06 | -0.02 | 0 | 0.92 | 0.078 | 1.1 | 0.95 |
| 5 | Polio_Duration | 0.17 | -0.05 | 0.05 | 0.94 | 0.05 | -0.02 | 0.1 | 0.93 | 0.0704 | 1.1 | 0.94 |
| 6 | Population_2020 | -0.08 | 0.05 | -0.33 | 0.01 | 0.04 | 0.07 | 0.65 | 0.55 | 0.4526 | 1.6 | 0.65 |
| 6 | Perc_Deaths_Hypertension | -0.34 | 0.02 | 0.27 | 0.02 | -0.11 | -0.28 | 0.47 | 0.5 | 0.4961 | 3.4 | 0.47 |
| 6 | Perc_Deaths_Diabetes | 0.02 | -0.14 | 0.26 | -0.04 | 0.03 | 0.06 | 0.33 | 0.2 | 0.7976 | 2.4 | 0.33 |
| 6 | Pop_Density | 0.05 | -0.14 | -0.07 | 0.14 | 0.11 | 0.04 | 0.27 | 0.13 | 0.8661 | 2.8 | 0.27 |
| 7 | Perc_Deaths_TB | -0.15 | 0.06 | 0 | 0.08 | 0.03 | 0.68 | -0.01 | 0.5 | 0.5022 | 1.1 | 0.68 |
| 7 | Perc_Deaths_HIV | -0.01 | 0.1 | -0.01 | -0.18 | 0 | 0.64 | -0.05 | 0.46 | 0.5396 | 1.2 | 0.64 |
| 7 | Perc_Deaths_Pneumonia | 0.18 | 0.11 | 0 | 0.12 | -0.04 | 0.46 | 0.22 | 0.32 | 0.6794 | 2.1 | 0.46 |

Supplementary Table 4: Average silhouette coefficient & Dunn Index for all clustering solutions. The Dunn Index and the average silhouette width indicate how similar an object is to its own cluster (cohesion) compared to other clusters (separation). Higher values indicate better clustering. Therefore, both a 6 and 9 country cluster were considered for country clustering.

| **Cluster separation metrics** | **5 cluster** | **6 cluster** | **7 cluster** | **8 cluster** | **9 cluster** | **10 cluster** |
| --- | --- | --- | --- | --- | --- | --- |
| Dunn Index | 0.18 | 0.32 | 0.28 | 0.28 | 0.33 | 0.29 |
| Average Silhouette Width | 0.31 | 0.33 | 0.31 | 0.31 | 0.32 | 0.30 |
| Average between distance | 3.64 | 3.62 | 3.53 | 3.52 | 3.52 | 3.48 |
| Average within distance | 1.96 | 1.84 | 1.74 | 1.67 | 1.61 | 1.55 |
| Within cluster SS | 116.20 | 99.87 | 88.65 | 78.57 | 69.19 | 62.50 |

Supplementary Table 5: The rate of missing data values and the treatment for each.

| **Factor** | **Variable** | **% Missing Values** | **Missing Value Treatment** |
| --- | --- | --- | --- |
| **Economic Factors** | GDP_Per_Capita | 0% | None |
| **Demographic Factors** | Population_2020 | 0% | None |
|  | Population Density | 0% | None |
|  | Percent_Pop_Above_65 | 0% | None |
|  | Percent_Pop_Above_80 | 0% | None |
|  | Household_Size_2019 | 19.30% | Mean imputation |
| **Geographic Factors** | Avg_Temp | 0% | None |
| **COVID-19 related** | Tests_Mn_30_Days_After_100th_Case | 0% | None |
|  | Deaths_Mn_30_Days_After_100th_Case | 0% | None |
| **BCG Immunization** | BCG_Immunization_Ever | 0% | None |
|  | BCG_Immunization_Current | 0% | None |
|  | BCG_Last_15_Coverage_Yes | 0% | None |
|  | BCG_Last_40_Coverage_Yes | 0% | None |
|  | BCG_Years_Of_Immunization | 0% | None |
|  | BCG_Coverage_Percentage | 5% | Imputed with a very large number (-100) to distinguish from cases with no BCG immunization |
|  | Perc_BCG_Coverage_GT50 | 5% | Taken as "Not recorded" and considered as a separate group |
|  | BCG_coverage_last_40yrs | 7% | Imputed with a very large number (-100) to distinguish from cases with no BCG immunization |
| **Rubella vaccine Coverage** | RCV1_Coverage | 0% | None |
|  | RCV1_Duration | 0% | None |
| **Measles vaccine coverage** | MCV1_Coverage | 16% | Imputed with a very large number (-100) to distinguish from cases with no BCG immunization |
|  | MCV1_Duration | 16% | Imputed with a very large number (-100) to distinguish from cases with no BCG immunization |
| **Polio vaccine coverage** | Polio_Coverage | 5% | Imputed with a very large number (-100) to distinguish from cases with no BCG immunization |
|  | Polio_Duration | 5% | Imputed with a very large number (-100) to distinguish from cases with no BCG immunization |
| **Disease Burden** | Perc_Deaths_Diabetes | 5% | Mean imputation |
|  | Perc_Deaths_Hypertension | 5% | Mean imputation |
|  | Perc_Deaths_Cerebrovascular | 5% | Mean imputation |
|  | Perc_Deaths_Pneumonia | 5% | Mean imputation |
|  | Perc_Deaths_Lower_Resp_Disease | 5% | Mean imputation |
|  | Perc_Deaths_TB | 5% | Mean imputation |
|  | Perc_Deaths_Obesity | 5% | Mean imputation |
|  | Perc_Deaths_HIV | 5% | Mean imputation |
| **Government measures** | Stringency_Index | 0% | None |
| **Efficiency of public health systems** | Overall_GHSA_Score | 0% | None |
|  | GHSA1_Emergence_Of_Disease | 0% | None |
|  | GHSA2_Detection | 0% | None |
|  | GHSA3_Response | 0% | None |
|  | GHSA4_Sufficient_System | 0% | None |
|  | GHSA5_Commitments | 0% | None |
|  | GHSA6_Risk | 0% | None |

Supplementary Table 6: Simplified composition of 9 clusters included for analysis.

|  | Cluster 1 | Cluster 2 | Cluster 3 | Cluster 4 | Cluster 5 | Cluster 6 | Cluster 7 | Cluster 8 | Cluster 9 |
| --- | --- | --- | --- | --- | --- | --- | --- | --- | --- |
| Number of Countries | 3 | 4 | 6 | 7 | 10 | 4 | 7 | 15 | 1 |
|  |  |  |  |  |  |  |  |  |  |
| GDP for Capita (lowest last) | 8 | 4 | 3 | 2 | 5 | 9 | 6 | 7 | 1 |
| % population > 65 y/o (lowest last) | 7 | 4* | 1 | 3 | 2 | 9 | 6 | 8 | 4* |
| Average Temperature (coldest last) | 1* | 5* | 5* | 9 | 7 | 1* | 8 | 3 | 4 |
| COVID testing/million at 30 cases after first 100 cases (lowest last) | 9 | 2 | 4 | 1 | 3 | 8 | 5 | 7 | 6 |
| Stringency Index (lowest late) | 7 | 4* | 9 | 4* | 3 | 1 | 6 | 2 | 8 |
| Overall GHSA score (lowest last) | 6 | 4 | 2 | 3 | 5 | 9 | 8 | 7 | 1 |
| Countries comprising each cluster | Brazil  Indonesia  Nigeria | Belgium  Israel  Netherlands  South Korea | Australia  France  Germany  Japan  Sweden  The United Kingdom | Austria  Canada  Denmark  Finland  Ireland  Norway  Switzerland | Czech Republic  Greece  Hungary  Italy  Poland  Portugal  Romania  Serbia  Spain  Ukraine | Bangladesh  Pakistan  Philippines  India | Afghanistan  Chile  Iran  Morocco  Turkey  Russia  Kazakhstan | Algeria  Cameroon  Colombia  Ecuador  Egypt  Ghana  Iraq  Malaysia  Mexico  Oman  Peru  Saudi Arabia  South Africa  Thailand  Uzbekistan | The United States of America |

Supplementary Table 7: Results of CSM compared within similarly clustering countries with and without a BCG program for either the preceding 15 or 40 years. At 40 preceding years, 7/9 clusters were evaluable. 5/7 clusters demonstrated decreased CSM. At 15 preceding years, 6/9 clusters were evaluable, and benefit was demonstrated in 4/6 clusters.

|  | Active program for Preceding 40 years | | | Active program for Preceding 15 years | | |
| --- | --- | --- | --- | --- | --- | --- |
| Cluster | Deaths/Million with BCG Vaccination | Deaths/Million without BCG Vaccination | HR | Deaths/Million with BCG Vaccination | Deaths/Million without BCG Vaccination | HR |
| 1 | 5.8 | 0.9 | 6.13 | 2.5 | NA | NA |
| 2 | 2.0 | 79.9 | 0.03 | 2.0 | 79.9 | 0.03 |
| 3 | 0.3 | 33.7 | 0.01 | 0.3 | 33.7 | 0.01 |
| 4 | 73.9 | 30.5 | 2.43 | 73.9 | 30.5 | 2.43 |
| 5 | 18.6 | 84.0 | 0.22 | 18.6 | 84.0 | 0.22 |
| 6 | 1.2 | NA | NA | 1.2 | NA | NA |
| 7 | 5.9 | 28.3 | 0.21 | 9.1 | NA | NA |
| 8 | 3.1 | 6.1 | 0.52 | 2.7 | 23.9 | 0.11 |
| 9 | NA | 17.9 | NA | NA | 17.9 | NA |

Supplementary Table 8a. Granular data regarding the composition of each cluster in the 6-cluster solution.

| **Cluster Number** | | **1** | **2** | **3** | **4** | **5** | **6** |
| --- | --- | --- | --- | --- | --- | --- | --- |
| **# Countries** | | **4** | **7** | **7** | **7** | **12** | **20** |
| **Economic Factors** | **GDP per capita** | 43,405 | 64,643 | 49,440 | 3,305 | 17,249 | 7,342 |
| **Demographic Factors** | **Population (mn)** | 22 | 11 | 101 | 361 | 35 | 49 |
|  | **Population Density** | 457 | 81 | 149 | 388 | 93 | 65 |
|  | **% pop above 65 yrs.** | 16.0% | 18.3% | 20.2% | 5.6% | 17.6% | 5.9% |
| **Geographic Factors** | **Average Temperature (Feb, Mar, Apr)** | 9 | 2 | 9 | 26 | 6 | 19 |
| **COVID related factors** | **Tests/mn (30 days after 100 cases)** | 9,304 | 13,727 | 5,731 | 277 | 8,325 | 2,163 |
| **Disease Burden** | **% Deaths - Diabetes** | 0.02% | 0.02% | 0.02% | 0.02% | 0.02% | 0.02% |
|  | **% Deaths - Hypertension** | 0.02% | 0.02% | 0.03% | 0.05% | 0.05% | 0.03% |
|  | **% Deaths - Cerebrovascular** | 0.04% | 0.05% | 0.05% | 0.02% | 0.10% | 0.02% |
|  | **% Deaths - TB** | 0.00% | 0.00% | 0.00% | 0.00% | 0.00% | 0.00% |
|  | **% Deaths - Pneumonia** | 0.02% | 0.02% | 0.03% | 0.03% | 0.02% | 0.02% |
|  | **% Deaths - Lower respiratory disease** | 0.03% | 0.03% | 0.02% | 0.02% | 0.04% | 0.01% |
|  | **% Deaths - Obesity** | 0.07% | 0.08% | 0.08% | 0.08% | 0.15% | 0.08% |
|  | **% Deaths - HIV** | 0.00% | 0.00% | 0.00% | 0.00% | 0.00% | 0.00% |
| **Government measures** | **Stringency Index** | 88 | 88 | 73 | 89 | 90 | 89 |
| **Efficiency of public health systems** | **Overall GHSA score** | 64 | 66 | 72 | 46 | 52 | 45 |

Supplementary Table 8b. Granular data regarding the composition of each cluster in the 9-cluster solution.

| **Cluster Number** | | **1** | **2** | **3** | **4** | **5** | **6** | **7** | **8** | **9** |
| --- | --- | --- | --- | --- | --- | --- | --- | --- | --- | --- |
| **# Countries** | | **3** | **4** | **6** | **7** | **10** | **4** | **7** | **15** | **1** |
| **Economic Factors** | **GDP per capita** | 4,948 | 43,405 | 47,214 | 64,643 | 18,589 | 2,073 | 7,967 | 7,478 | 62,795 |
| **Demographic Factors** | **Population (mn)** | 236 | 22 | 63 | 11 | 26 | 455 | 61 | 47 | 331 |
|  | **Population Density** | 129 | 457 | 168 | 81 | 111 | 582 | 48 | 65 | 36 |
|  | **% pop above 65 yrs.** | 6.2% | 16.0% | 20.9% | 18.3% | 19.0% | 5.2% | 8.0% | 5.6% | 16.0% |
| **Geographic Factors** | **Average Temperature (Feb, Mar, Apr)** | 26 | 9 | 9 | 2 | 8 | 26 | 7 | 21 | 11 |
| **COVID related factors** | **Tests/mn (30 days after 100 cases)** | 157 | 9,304 | 6,027 | 13,727 | 7,924 | 367 | 4,963 | 1,946 | 3,957 |
| **Disease Burden** | **% Deaths - Diabetes** | 0.02% | 0.02% | 0.02% | 0.02% | 0.02% | 0.02% | 0.02% | 0.02% | 0.02% |
|  | **% Deaths - Hypertension** | 0.05% | 0.02% | 0.03% | 0.02% | 0.05% | 0.05% | 0.03% | 0.03% | 0.06% |
|  | **% Deaths - Cerebrovascular** | 0.02% | 0.04% | 0.05% | 0.05% | 0.12% | 0.02% | 0.03% | 0.02% | 0.00% |
|  | **% Deaths - TB** | 0.00% | 0.00% | 0.00% | 0.00% | 0.00% | 0.01% | 0.00% | 0.01% | 0.00% |
|  | **% Deaths - Pneumonia** | 0.03% | 0.02% | 0.03% | 0.02% | 0.02% | 0.03% | 0.02% | 0.02% | 0.02% |
|  | **% Deaths - Lower respiratory disease** | 0.02% | 0.03% | 0.02% | 0.03% | 0.03% | 0.02% | 0.03% | 0.01% | 0.02% |
|  | **% Deaths - Obesity** | 0.08% | 0.07% | 0.08% | 0.08% | 0.16% | 0.08% | 0.09% | 0.07% | 0.09% |
|  | **% Deaths - HIV** | 0.00% | 0.00% | 0.00% | 0.00% | 0.00% | 0.00% | 0.00% | 0.00% | 0.00% |
| **Government measures** | **Stringency Index** | 79 | 88 | 73 | 88 | 90 | 95 | 81 | 93 | 76 |
| **Efficiency of public health systems** | **Overall GHSA score** | 51 | 64 | 70 | 66 | 53 | 41 | 44 | 45 | 84 |

Supplementary Figure 1: Correlation matrix assessing the intervariable association between pre-determined variables on COVID-19 related morality. Positive correlation is represented by positive numbers (blue) and negative correlation is represented by negative numbers (red). The size of the circle in each cell represents the magnitude of correlation. The figure is original to the authors and was creating using R Studio for windows (1.3.1093). URL - <https://rstudio.com/> & R for windows (4.0.3). URL - <https://www.r-project.org/>.


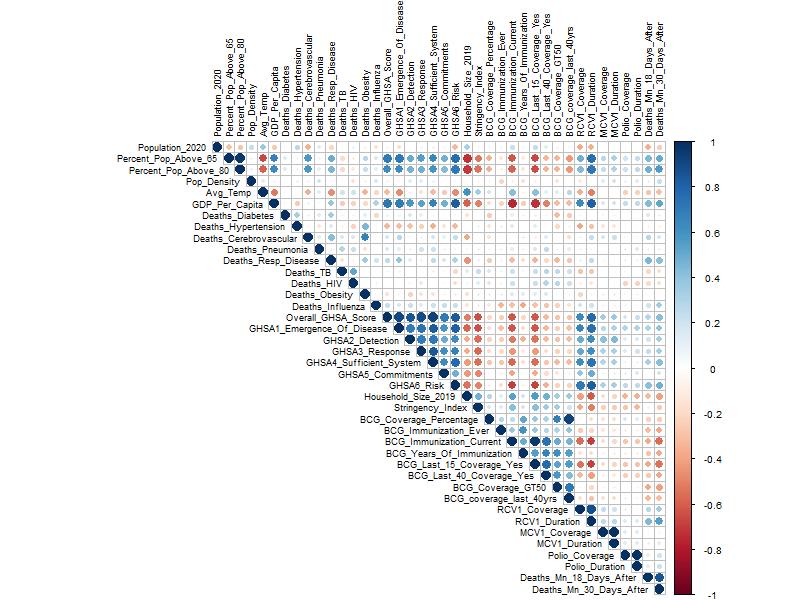


Supplementary Figure 2: Scree plot for determining optimum number of factors for analysis. Seven factors provided the optimum number of factors to include. The figure is original to the authors and was creating using R Studio for windows (1.3.1093). URL - <https://rstudio.com/> & R for windows (4.0.3). URL - <https://www.r-project.org/>


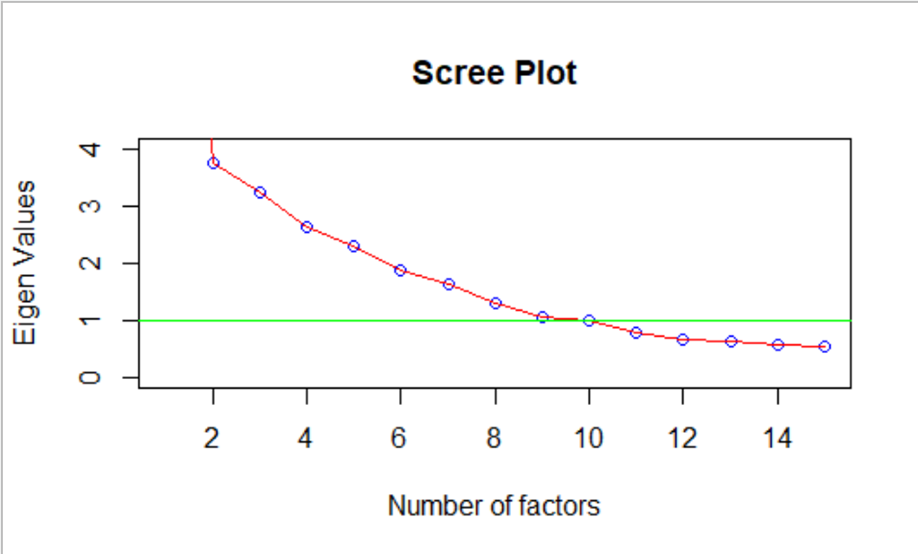


Supplementary Figure 3: Results of K-means analysis identifying the optimal number of clusters. Optimization was similar for inclusion of 6 or 9 clusters. The figure is original to the authors and was creating using R Studio for windows (1.3.1093). URL - https://rstudio.com/ & R for windows (4.0.3). URL - https://www.r-project.org/.


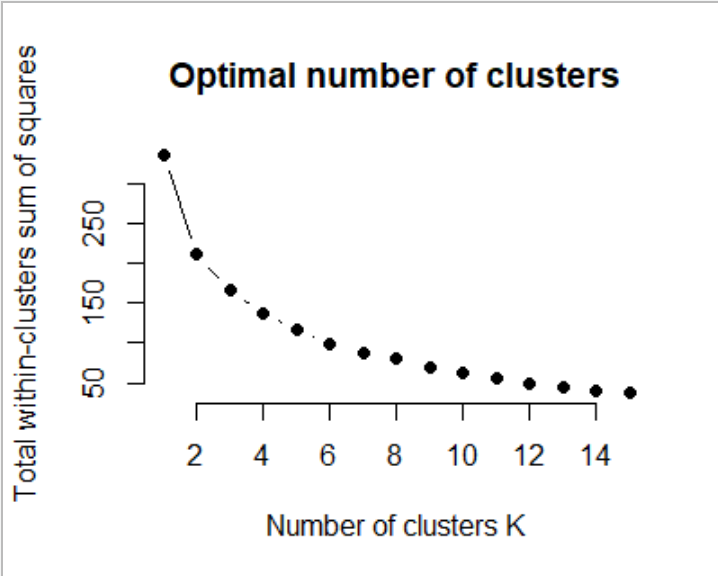

Supplement: Supplementary file 1 — Supplementary Information. [file 41598_2020_80787_MOESM1_ESM.docx]
